# Human behaviour directs household-level exposure to malaria vectors in Bandarban, Bangladesh

**DOI:** 10.1186/s12936-022-04375-4

**Published:** 2022-11-29

**Authors:** Matthew A. Aubourg, Hasan Mohammad Al-Amin, Anoop Sunkara, Sanjna Chetan, April Monroe, Ching Swe Phru, Rashidul Haque, Wasif A. Khan, Allison Hendershot, Mohammad Shafiul Alam, Neil F. Lobo

**Affiliations:** 1 Department of Biological Sciences, 321 Galvin Life Science Center, University of Notre Dame, Notre Dame, IN 46556 USA; 2 International Centre for Diarrheal Disease Research Bangladesh (icddr,b), Dhaka, Bangladesh; 3 QIMR Berghofer Medical Research Institute, Queensland, Australia; 4 Johns Hopkins Center for Communication Programs, Baltimore, MD USA

**Keywords:** Malaria, Human behaviour, Vector behaviour, Household exposure, Vector control, Spatiotemporal analysis, Residual transmission, Bangladesh, Chittagong Hill Tracts

## Abstract

**Background:**

Bangladesh has reduced malaria incidence and mortality by over 75% between 2010 and 2020. Widespread long-lasting insecticidal net (LLIN) distribution and use is one of the measures responsible for this success. Recalcitrant malaria hotspots within the Chittagong Hill Tracts districts suggest important drivers of malaria risk may remain uncharacterized.

**Methods:**

Towards understanding how household-level human behaviour impacts exposure to mosquitoes, parallel human landing catches and human behavioural observations were conducted in four households for 6 months (May–October) over the rainy season in the Bandarban District. Analysis quantifies spatiotemporal human behaviour-adjusted exposure to *Anopheles* with and without LLINs.

**Results:**

This small-scale operational study demonstrates that human spatial and temporal presence along with LLIN use drives exposure to *Anopheles*. Though the four households had both outdoor and indoor exposure, especially in the evening (1800–2000 h) and early morning (0400–0500 h), data points to household-based heterogeneity in malaria exposure even with similar LLIN access.

**Conclusion:**

Incorporating human behaviour into exposure estimates can be used to understand the efficacy and limitations of local vector control strategies and identify gaps in protection, as well as where present intervention strategies may be optimized.

## Background

**Fig. 1:**
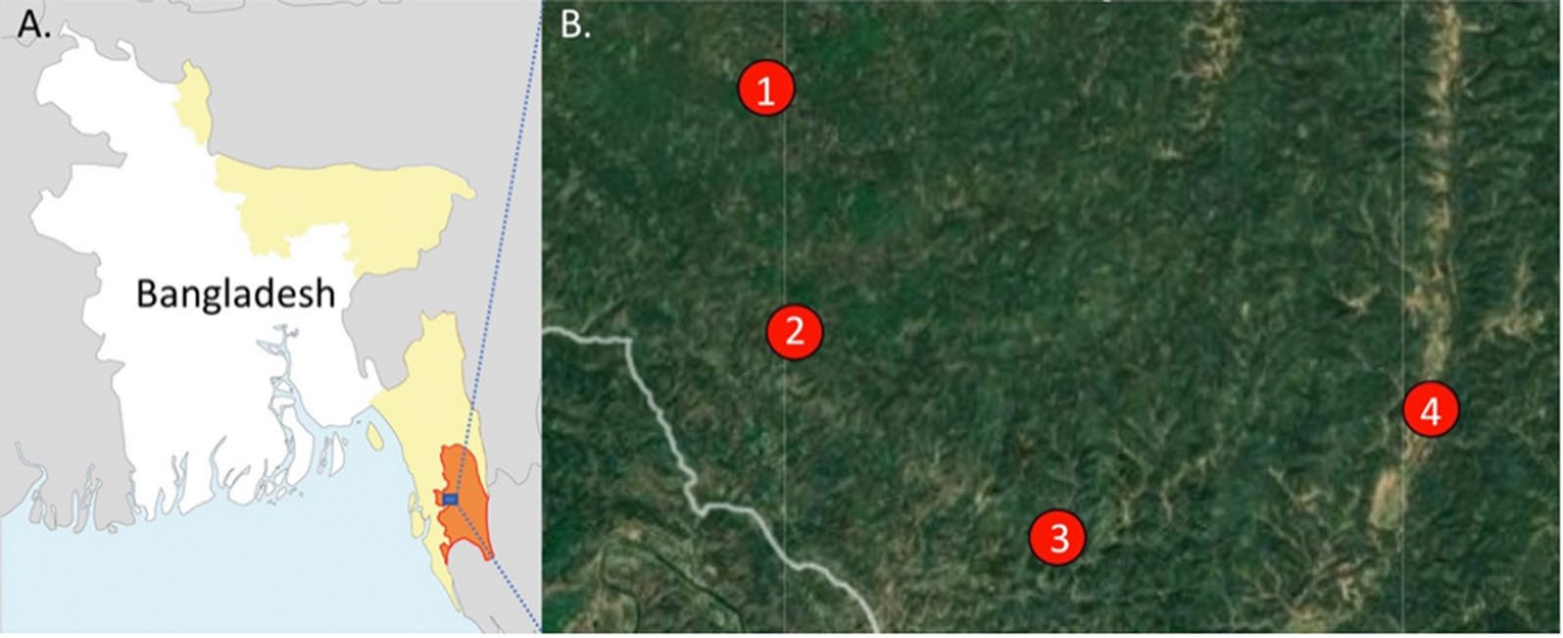
** A** Map of Bangladesh and bordering India and Myanmar. Areas of high malaria transmission highlighted in yellow. Bandarban District (orange) and the **B** collection sites are labelled. **B** The study sites are (1) Noa Para, (2) 1,2,3, Rubber Bagan, (3) Prue Mong U Headman Para, and (4) Jogesh and Chikka Para

Malaria remains a public health issue in Bangladesh; however, the disease has largely been eradicated in the majority of the country with a very low incidence rate of 1.2 per 1000 population in 2019 and a 63% decrease in total reported cases between 2017 and 2018 [1]. With 21,146 malaria cases in 2019, malaria transmission persists primarily, but not solely, in 13 of 64 districts, accounting for over 17 million people or around 11% of the population at risk [1, 2]. Cox’s Bazar and the three Chittagong Hill Tracts (CHT) districts (Bandarban, Khagrachari and Rangamati), which lie in the southeastern region of the country bordering India and Myanmar (Fig. 1), account for over 90% of total malaria cases and 80% of malaria-related deaths [3].

The region’s tropical climate and forested, mountainous topography maintain high *Anopheles* vector species diversity and cross-border transmission [4]. Refugee movement from neighboring malaria-endemic countries, India and Myanmar, complicate understanding and, therefore, control, of malaria transmission [5–7]. The high concentration of cases in these outer regions demands new, creative strategies tailored to the local drivers of residual malaria transmission.

The hyperendemic nature of malaria in the forested eastern border regions of Bangladesh solidifies the disease as a significant concern despite a low countrywide incidence rate. Countrywide malaria incidence and mortality has generally decreased between 2010 and 2019, concurrent with an increase in large-scale distribution of interventions such as rapid diagnostic testing and long-lasting insecticidal nets (LLINs), the primary malaria vector control intervention [1, 3, 8]. However, case numbers increased from 2018 to 2019 with a continued high malaria burden in the CHT districts where transmission is historically high [1]. Persistent transmission may be attributed to multiple factors including intervention coverage and usage, as well as the prevalence of high *Anopheles* diversity [4, 9, 10] and insecticide resistance [11] in these regions.

Since interventions take advantage of susceptible vector bionomic traits, species-specific population and bionomic data on local vectors are typically utilized when developing a baseline understanding of entomological drivers of malaria. However, humans may also drive intervention-vector overlap based on their spatial and temporal presence and intervention usage [12, 13]. Even in the early 1960s, researchers identified spatiotemporal interactions between humans and vectors as critical to transmission dynamics, including time spent indoors and outdoors [14]. More recent studies have expanded these considerations to also include use of preventive interventions at different periods of the day [12, 15–17]. Human behaviour-adjusted vector exposure values reveal the limitations of LLINs and provide the opportunity to create solutions targeting these gaps in protection, while characterizing potential transmission within times and spaces where humans and vectors overlap.

The increasing number of malaria cases observed from 2018 to 2019, a lack of knowledge of local entomological drivers of transmission, and potential gaps in protection can pose a challenge to the elimination agenda in Bangladesh. This small-scaled study evaluated bionomic characteristics of *Anopheles* species in relation to human behaviour and interventions towards understanding how household level behaviours might impact exposure (with community-wide vector control strategies) at four sentinel households in the Bandarban district.

## Methods

### Site description

This operational research study (part of a parent study evaluating *Anopheles* bionomics) was conducted in four villages (locally known as paras) within two unions (Kuhalong and Rajbila) in northern Bandarban, a tropical district in south-eastern Bangladesh (21°48′ N 92°24′ E) with one sentinel household per village (four total participating households) (Fig. 1). Bandarban is a zila (district) in the CHT [18] with a total area of 4479.01 sq. km. (1729.35 sq. miles), of which a large proportion is forested. The annual average temperature ranges from 13 to 34.6 °C in the winter and summer months. The rainy season is from the end of May to October with an average precipitation of 3031 mm per year [19, 20].

According to the 2011 national census, Kuhalong has a population of 11,075 and Rajbila has a population of 9123 [18]. Geographically, the two unions are adjacent to each other but have varying environments; Kuhalong is forested and hilly whereas Rajbila has more rice cultivation. Under the two unions, samples were collected from four sites: Rubber Bagan 1,2,3, Noa Para, Prue Mong U Headman Para, and Jogesh and Chikka Para. Structures in these sites were selected based on high malaria endemicity, elevation, and land use for agriculture. Crop cultivation sites included rubber plantations, rice fields, and ‘*jhum*’ sites (slash and burn cultivation).

Rubber Bagan 1,2,3 has 68 households and is surrounded by rubber plantations which are ecologically different from other sites based on monoculture. Here, sap collection cups are placed on rubber trees (*Hevea brasiliensis*) that collect rainwater and provide potential larval sites of various *Anopheles* species, such as *Anopheles vagus* [10]. Jogesh Para & Chikka Para, the only site in Rajbila Union, has a total of 89 households situated in several adjacent house clusters (villages). Noa Para has 41 households and two small cattle sheds, and is unique for its many rice fields, adjacent *jhum* cultivation areas, and a water canal at its northern limit. The community comprises mainly farmers and agriculturalists working in *jhum* sites. Prue Mong U Headman Para has 55 households at high elevation (105 m) atop hills. The lowland areas surrounding the hilly regions consist of rice fields to the east and dams retaining runoff water.

In the text and figures, each household is referred to by the corresponding village name, however, data presented only represents individual household level information.

### Human landing collections (HLCs)

Mosquito collections were conducted via overnight (1800–0600 h) paired indoor and outdoor human landings collections (HLCs) across structures that represented typical household construction and number of inhabitants. Representative household structures were built with wood and bamboo with thatch or tin roofs, with open eaves and porous walls and floors. Most households were 3–4 rooms in size with structures being elevated slightly above the ground. Cattle were usually kept close by in cattle pens with chickens and other smaller domestic animals living below or around the households. There were four to eight inhabitants per household representing families. One structure was selected at each site and data collection was conducted for two to four nights per month from May to October (2018) during the monsoon season. *Anopheles* landing rates were used as a proxy for human biting rates (HBR—bites/person/night (bpn) or bites/person/hour (bph)). When no mosquitoes were collected during a particular hour, a man biting rate of 0.01 bph was assumed to reflect the real-world possibility of mosquito biting.

### Sample processing

*Anopheles* mosquitoes were identified morphologically [21, 22] and stored with desiccant in labeled (collection hour, location, morphological identity, and house code) 1.5 mL microcentrifuge tubes.

### Human behaviour

Human behaviour observations (HBOs) [12, 13] were conducted parallel and concurrently to HLCs towards matched household human and vector data. International Centre for Diarrheal Disease Research, Bangladesh (icddr,b) staff observed and recorded the number of household members in each behavioural category [(a) inside awake without LLINs, (b) inside awake with LLINs, (c) inside asleep without LLINs, (d) inside asleep with LLINs, and (e) outdoors (awake or asleep)] at the end of each HLC hour in each location of HLC collection (inside or outside). HLC collectors were not household members. Data was collected on paper forms, with a supervisor performing spot checks for quality control. Data was entered into Excel and compared to the paper forms for accuracy.

### Human behaviour-adjusted biting rates (HBBRs)

Human behaviour-adjusted biting rates (HBBRs) were generated using HBRs and HBOs [12] in the following categories: (a) behaviour-adjusted bites occurring indoors awake, (b) behaviour-adjusted bites occurring indoors asleep (LLINs not used), (c) behaviour-adjusted bites occurring outdoors, and (d) behaviour-adjusted bites prevented by using LLINs.

## Results

Vector landing rates inside and outside households, the spatial and temporal presence of humans, as well as LLIN usage and human behaviours were characterized at the four sentinel houses for 6 months.

### Vector species composition and behaviour

Human landing catches allowed for descriptions of HBRs over the wet season (May to October). Female *Anopheles* mosquitoes (n = 96) were collected between May and October at all four structures using HLCs. These specimens consisted of 14 species—the three most common species included *Anopheles maculatus*, *An. vagus*, and *Anopheles baimaii*, while lesser common species included *Anopheles jeyporiensis*, *Anopheles peditaeniatus*, *Anopheles barbirostris, Anopheles varuna, Anopheles kochi, Anopheles nivipes, Anopheles karwari, Anopheles jamesii, Anopheles philippinensis, Anopheles umbrosus, Anopheles campestris*, and *Anopheles nigerrimus*. The primary indoor biting species were *An. vagus* (20.5% of indoor collections) and *An. maculatus* (13.6%), while *An. maculatus* (17.3% of outdoor collections), *An. jeyporiensis* (15.4%), *An. baimaii* (13.5%), *An. peditaeniatus* (9.6%), and *An. vagus* (9.6%) were the primary outdoor biting species (Rodriguez, in preparation).

At all sites, both indoor and outdoor landing rates peaked between July and October—2 months after peak rainfall. Both indoor and outdoor biting rates tended to be highest in the early evening and early morning (for example, Rubber Bagan 1,2,3 had peaks at 1900 h with 1.5 bph, and at 0400 h with 2.33 bph), however, there was wide variability in hourly biting rates per site.

**Fig. 2:**
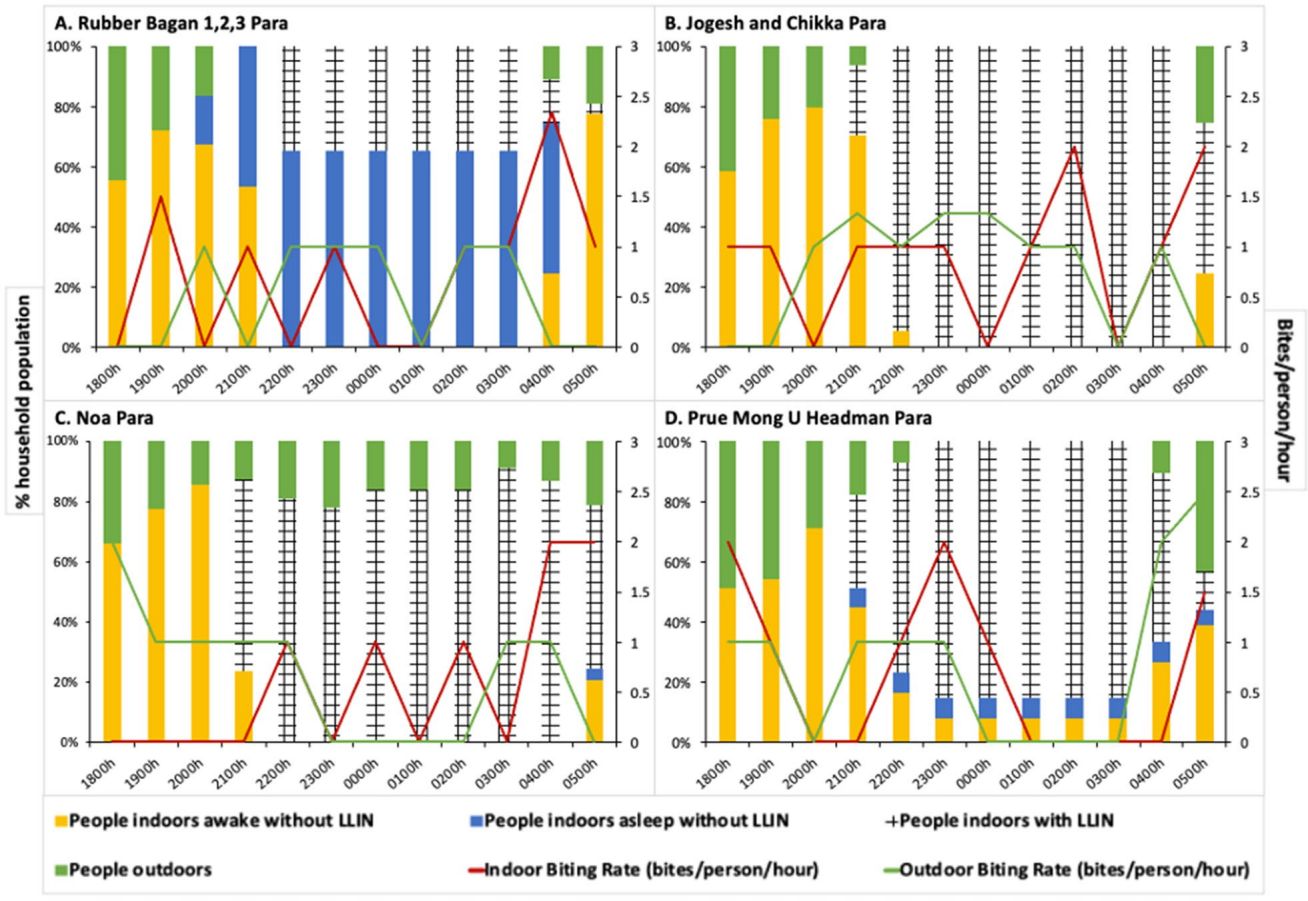
Human behaviours and vector biting rates for each household. Proportions of people in four behavioural categories—outdoors (green), indoors using LLINs (crosshatch), indoors awake without LLINs (yellow), and indoors asleep without LLINs (blue)—are depicted in the bar graphs for the four households in: **A** Rubber Bagan 1,2,3, **B** Jogesh and Chikka Para, **C** Noa Para and **D** Prue Mong U Headman Para. Parallel indoor (red) and outdoor (green) vector biting rates are illustrated with line graphs. People tend to be active in the evening to night (1800–2100 h) and in the early morning (0400–0500 h). Household-specific differences in human behaviour are seen. Low LLIN use is observed in Rubber Bagan 1,2,3 while high usage of LLINs is seen in other households, beginning at 2100 h

The Rubber Bagan 1,2,3 Para household experienced an overall *Anopheles* HBR of 1.34 bpn indoors and 0.67 bpn outdoors. Jogesh and Chikka Para reported a similar indoor HBR of 1.33 bpn and a higher outdoor HBR of 1.75 bpn. The lowest HBRs of 0.59 bpn indoors and 0.91 bpn outdoors were determined at Noa Para, while Prue Mong U Headman experienced rates of 0.92 bpn indoors and 1.25 bpn outdoors. Indoor and outdoor biting rates remained consistent throughout the night (Fig. 2).

### Human behaviour observations

All four households demonstrated a degree of household-based heterogeneity in human behaviour across time and space. Generally, people moved indoors from the start of data collection (1800 h), with LLIN usage beginning at 2100 h, and most people in bed by 2200 h (Fig. 2). People generally woke up and moved outdoors from 0400 to 0500 h onwards.

In Rubber Bagan 1,2,3, two to seven household members were observed sleeping without bed nets across nearly all months of collection, at times at nearly twice the proportion of bed net users. Indoor *Anopheles* biting peaks coincided with periods where most people were indoors not using bed nets. On the other hand, the other three households had high LLIN use.

The Jogesh and Chikka Para household demonstrated high LLIN usage (94.1 to 100%) during sleeping hours (2200–0400 h) which overlapped with higher vector indoor biting rates. This household had no outdoor sleeping behaviours documented during this period.

At Noa Para, a proportion of people remained outdoors over the night. Outdoor biting rates were highest (2 bph) when the proportion of people outdoors was the highest (33.3%) at 1800 h. Indoor biting rates were high (1–2 bph) during sleeping hours when most people were under LLIN protection (78.6–91.7%), while higher indoor biting rates coincided with people waking up.

The Prue Mong U Headman Para household demonstrated the greatest number of people outdoors at 1800, 1900, and 0500 h—all periods that coincided with high outdoor biting rates. The highest outdoor biting rates coincided with people moving outdoors from 0400 h onwards. Indoor biting rates were also high at those times with increasing proportions of people active indoors. Though a proportion of the household used LLINs while asleep indoors at these times, a small proportion (5.36% at 0400 and 0500 h) did not. Note that LLIN-use was not observed outdoors.

### Human behaviour-adjusted biting rates

**Fig. 3:**
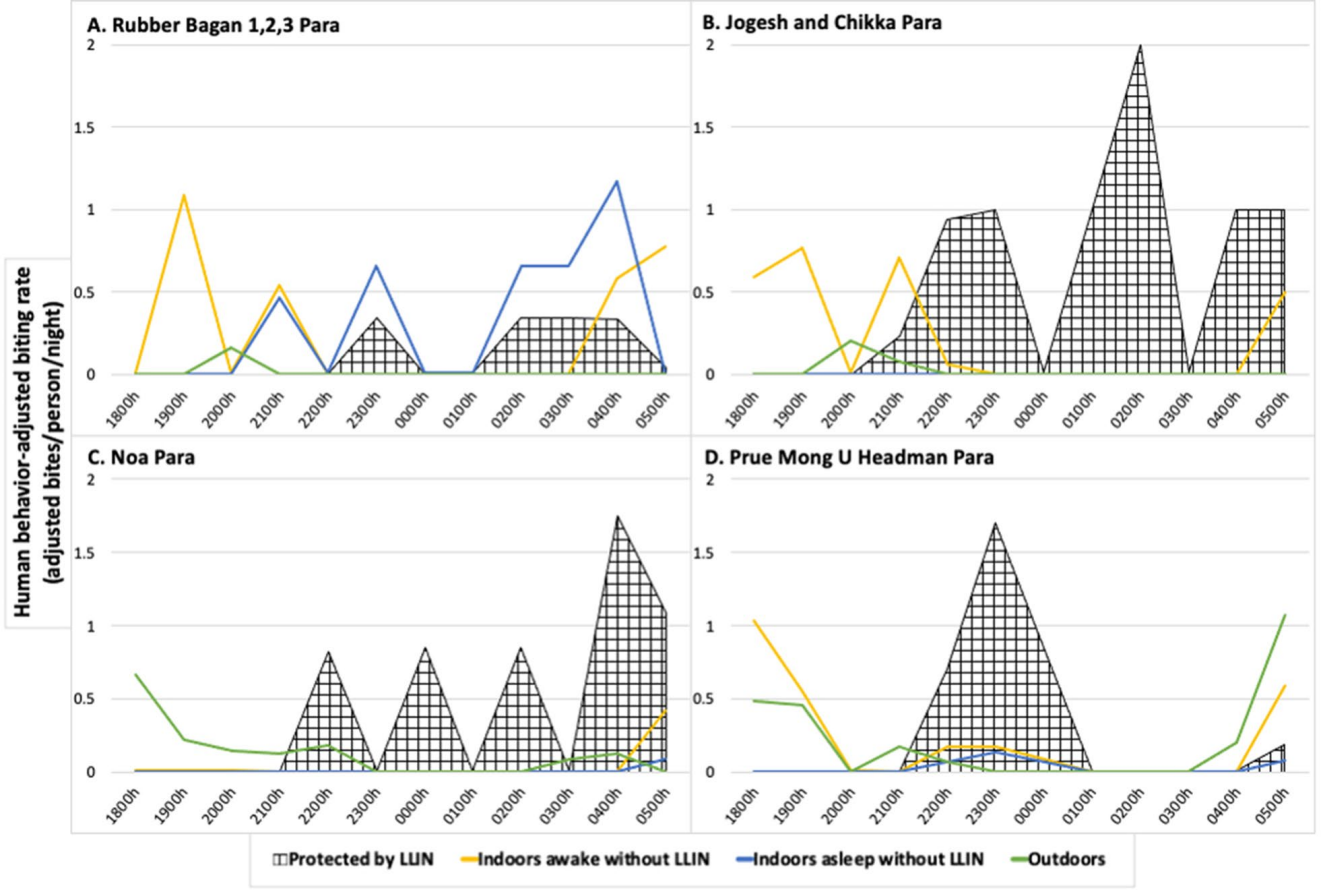
Human behaviour-adjusted biting rates for each household. Human behaviour-adjusted biting rates (HBBRs) per hour at each structure in **A** Rubber Bagan 1,2,3, **B** Jogesh and Chikka, **C** Noa, and **D** Prue Mong U Headman Paras. Protection by LLIN (crosshatch) is found at all sites. Exposure awake indoors without LLINs (yellow) and outdoors (green) is greatest in the early evening and early morning at all sites, and exposure while asleep without LLIN use (blue) is low at all sites except at the household in Rubber Bagan 1,2,3 Para which is also characterized by low LLIN use

**Fig. 4:**
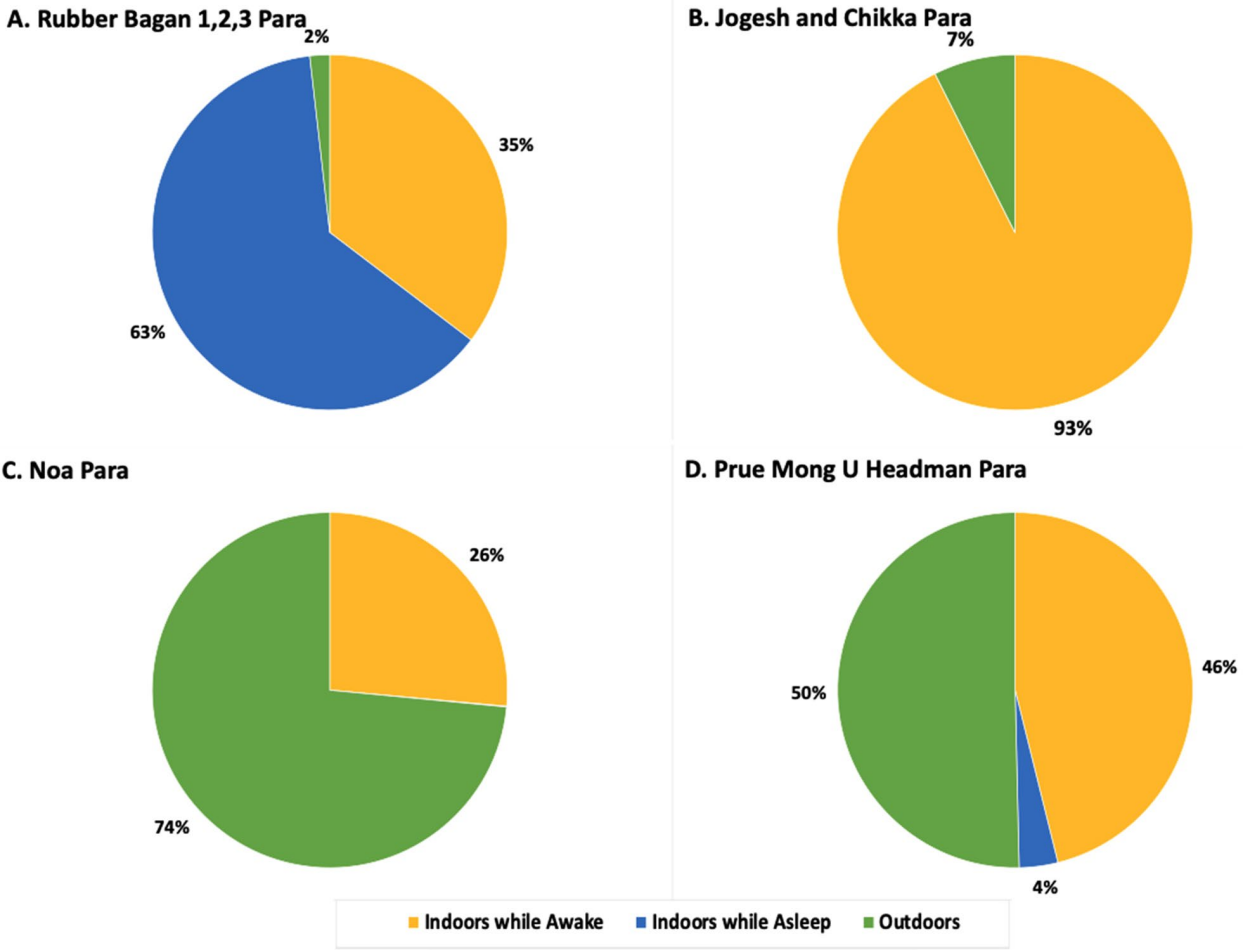
Proportions of exposure to mosquito bites at each household based on human behaviour-adjusted biting rates. Human behavioural observations in combination with vector biting rates were used to determine exposure at each household—**A** Rubber Bagan 1,2,3, **B** Jogesh and Chikka Para, **C** Noa Para, and **D** Prue Mong U Headman Para. Significant exposure occurs indoors when asleep (blue) due to lower LLIN use in the household at Rubber Bagan 1,2,3. Outdoor exposure (green) is demonstrated in Noa Para and Prue Mong U Headman Para households, while all sites have some level of exposure indoors when awake without LLIN protection (yellow)

Human behaviour-adjusted biting rates (HBBRs), determined from HBO and HLC data, outline spatial and temporal site-specific exposure (Figs. 3 and 4). The Rubber Bagan 1,2,3 household was the only structure with a majority (HBBR = 4.07 adjusted bpn, 63%) of exposure while indoors asleep with little outdoor exposure (0.12 adjusted bpn, 2%). The Jogesh and Chikka Para household demonstrated higher exposure while indoors and awake (1.80 adjusted bpn, 93%) and low outdoors exposure (0.15 adjusted bpn, 7%). At Noa Para, household outdoor exposure was higher (1.34 adjusted bpn, 74%) with some exposure while indoors and awake (0.48 adjusted bpn, 26%). HBBRs depicted exposure at the Prue Mong U Headman Para household in all behavioural categories: outdoors exposure at 2.64 adjusted bpn (50%), awake indoors at 2.42 adjusted bpn (46%), and indoors asleep without LLIN protection at 0.18 adjusted bpn (4%). LLIN-based protection from mosquito bites was documented at each household based on observed LLIN use.

## Discussion

Successful malaria intervention strategies in Bangladesh have brought the country close to malaria elimination. However, persistent malaria transmission even with blanket LLIN coverage within the southeastern outer regions requires renewed evaluation to determine the possible drivers of transmission. The present study examined paired HLCs and HBOs to characterize vector species behaviours alongside human behaviours and determine the spatiotemporal and behavioural drivers that influence exposure to malaria vector biting on a household level.

### Seasonality and drivers of transmission

Human landing catches and HBOs were collected from May to October, spanning the monsoon, or malaria transmission season (June to mid-August). Higher early monsoon rainfall in these hilly areas may wash out larval sites resulting in the reduction of adult populations and idling malaria transmission [23]. Increasing mosquito biting rates later (June and July) in the monsoon observed here were associated with lower rainfall and persistent larval sites. The increased biting rates may be linked with greater observed malaria incidence in Bandarban following the primary rains [24].

The high diversity of mosquitoes found at this site with an associated spectrum of bionomic characteristics may result in the transmission seen here even with lower HBRs. Multiple and variable species-specific biting behaviours would take advantage of specific and variable spatiotemporal points where humans are without protection from bites. Higher densities of mosquitoes present were documented by other sampling methods used in another part of the parent study.

### Vector species composition

Fourteen different previously documented [4] *Anopheles* species were collected across all four sites in HLC collections. *Anopheles vagus* was the predominant species which also has long been identified as a primary malaria vector in Bangladesh with high rates of *Plasmodium* infection and found alongside other primary vectors [4, 9, 25]. Recently, it has been identified as a major threat to malaria interventions in Bangladesh due to rising levels of insecticide resistance [11]. Therefore, with abundant *An. vagus*, there is greater urgency to consider spatiotemporal and behavioural contexts, such as the focus of this study, in targeting malaria interventions. Another important species collected both indoors and outdoors was *An. baimaii*—which is considered a principal malaria vector in this region [26, 27]. The high number of species present at this site with corresponding differential behaviours points to species-specific temporal and spatial exposure over the course of a night as well as possibilities for selection of bionomic traits that enable them to avoid interventions and continue transmission.

### Overlapping vector and human behaviours

All households pointed to a similar general picture of human behaviour: people move indoors to be active without LLINs in the evening (1800–2000 h), sleep during the night (2100–0400 h), and then gradually wake up to be active indoors or outdoors in the early morning (0400–0500 h). These human behaviours—in conjunction with interventions present and in use—and mosquito behaviours dictate the intensity of human-vector contact. Paired household data suggest that current use of LLINs along with gaps in protection, revealed by overlapping vector and human behaviours, may greatly differ across households driving household malaria risk.

The household in Rubber Bagan 1,2,3 had low LLIN use (Fig. 2A) with 65.5% of household members sleeping without LLIN protection between 2200 and 0300 h; the greatest proportion of individuals within this same behavioural category at other households was 6.78%. Household members reported that low LLIN use is attributed to high humidity and heat-related discomfort associated with sleeping under an LLIN in this site that lacks electricity. At the same time, indoor biting rates peaked at 0400 h (2.33 bph) where 50% of individuals were sleeping without LLINs and 25% were active indoors—behaviours resulting in increased exposure and a gap in protection. Increased LLIN use, possibly accomplished with social and behaviour change interventions, would enable greater protection [28] and optimize the current bed net-based intervention strategy in communities with similar behaviours.

The households in Jogesh and Chikka Para and Noa Para, on the other hand, greatly minimized gaps in protection through the night (2200–0500 h) with high proportions of people sleeping under LLINs when indoor biting rates were highest (Fig. 2B, C). However, outdoor presence during the night without LLIN protection at the household in Noa Para coincided with higher outdoor biting rates and represent a gap in protection that cannot be addressed by LLIN use. *Jhum* cultivation practices are likely responsible for this outdoor nighttime exposure where cultivators were guarding the adjacent *jhum* area [29, 30]. Exposure to bites indoors when awake, present in all houses, also suggest a gap in protection that cannot be addressed by LLIN use. Interventions such as spatial and topical repellents may be of use in lowering exposure to biting [31–34].

At the household in Prue Mong U Headman Para, though most individuals indoors were LLIN-protected during the night (Fig. 2D), some people remained indoors unprotected while sleeping and active at times of higher indoor biting rates. Outdoor exposure also increased towards the early morning as more people moved outdoors. Overlapping vector and human activity in the evening (1800–1900 h) also highlight gaps in protection both indoors and outdoors with high indoor and outdoor biting rates and high proportions of household members active outside and inside.

Interestingly, outdoor HLC biting rates were usually higher during the night when most individuals were sleeping indoors (at all households) suggesting that HLC-based biting rates may not actually represent exposure to mosquito bites [12].

## Limitations and broader implications

This study presents data from only four households over six months. Since HBOs and mosquito collections were conducted in an operational context at a single household per site, the variability and range of human and vector behaviours over the population were not captured. Budget constraints with the parent study did not allow for the inclusion of a population-based, statistically viable sample size. This study also did not capture occupational or mobility-based risk and time spent away from the primary household. Mosquito sampling in extra-domestic *jhum* cultivation areas demonstrated high vector numbers and represent additional community exposure. Though this study demonstrates that household level variation in human behaviour can direct exposure, a larger dataset consisting of population level human and mosquito data would more accurately define intervention needs.

Other drivers of mosquito populations that require further investigation include the presence of *jhum*, rice, and rubber cultivation areas, and water dams which also drive mosquito biting rates [29, 35–41] even by a factor of 12 times more than non-irrigated areas [42].

## Conclusion

The present study is the first proof of principle operational study to evaluate parallel HLCs and HBOs to develop adjusted exposure rates in Bangladesh on a household level. Data suggests that human behaviour on a household level can impact exposure to malaria vectors even with recommended community-wide LLIN implementation. A population-level understanding of how human behaviours interact with interventions and vector behaviours will enable the estimation of community-wide spatial and temporal exposure as well as point to how LLIN-based strategies may be optimized with these populations.

## Data Availability

The datasets supporting the conclusions of this article are available from the corresponding author upon reasonable request.

## Abbreviations

LLIN: Long-lasting insecticidal net
CHT: Chittagong Hill Tracts
HLC: Human landing catches
HBO: Human behaviour observations
bpn: Bites per person per night
bph: Bites per person per hour
HBBR: Human behaviour-adjusted biting rate
